# Hepatitis C Virus NS5A Inhibits Mixed Lineage Kinase 3 to Block Apoptosis*

**DOI:** 10.1074/jbc.M113.491985

**Published:** 2013-07-15

**Authors:** Yutaka Amako, Zsofia Igloi, Jamel Mankouri, Arunas Kazlauskas, Kalle Saksela, Mark Dallas, Chris Peers, Mark Harris

**Affiliations:** From the ‡ School of Molecular and Cellular Biology, Faculty of Biological Sciences and; the ¶ Division of Cardiovascular and Diabetes Research, Faculty of Medicine and Health, University of Leeds, Leeds LS2 9JT, United Kingdom and; the § Department of Virology, Haartman Institute, University of Helsinki Central Hospital, University of Helsinki and HUSLAB, FI-00014 Helsinki, Finland

**Keywords:** Apoptosis, Hepatitis c Virus, Ion Channels, Oxidative Stress, SH3 Domains, Kv2.1, NS5A, Mixed Lineage Kinase 3

## Abstract

Hepatitis C virus (HCV) infection results in the activation of numerous stress responses including oxidative stress, with the potential to induce an apoptotic state. Previously we have shown that HCV attenuates the stress-induced, p38MAPK-mediated up-regulation of the K^+^ channel Kv2.1, to maintain the survival of infected cells in the face of cellular stress. We demonstrated that this effect was mediated by HCV non-structural 5A (NS5A) protein, which impaired p38MAPK activity through a polyproline motif-dependent interaction, resulting in reduction of phosphorylation activation of Kv2.1. In this study, we investigated the host cell proteins targeted by NS5A to mediate Kv2.1 inhibition. We screened a phage-display library expressing the entire complement of human SH3 domains for novel NS5A-host cell interactions. This analysis identified mixed lineage kinase 3 (MLK3) as a putative NS5A interacting partner. MLK3 is a serine/threonine protein kinase that is a member of the MAPK kinase kinase (MAP3K) family and activates p38MAPK. An NS5A-MLK3 interaction was confirmed by co-immunoprecipitation and Western blot analysis. We further demonstrate a novel role of MLK3 in the modulation of Kv2.1 activity, whereby MLK3 overexpression leads to the up-regulation of channel activity. Accordingly, coexpression of NS5A suppressed this stimulation. Additionally we demonstrate that overexpression of MLK3 induced apoptosis, which was also counteracted by NS5A. We conclude that NS5A targets MLK3 with multiple downstream consequences for both apoptosis and K^+^ homeostasis.

## Teaser

**Background:** Oxidative stress is a key event in the determination of host cell fate.

**Results:** The hepatitis C virus NS5A protein binds to the MLK3 kinase, blocking both oxidative stress induced apoptosis and activation of the potassium channel Kv2.1.

**Conclusion:** NS5A blocks host cell apoptosis and potassium efflux.

**Significance:** Interference with the host stress response could facilitate viral persistence.

## Introduction

Hepatitis C virus (HCV)^4^ is a highly pathogenic human virus associated with liver fibrosis, steatosis, and cancer (1). HCV is an enveloped virus belonging to the Hepacivirus genus of the family Flaviviridae. The HCV genome comprises a 9.6 kb RNA with sense strand polarity containing a single open reading frame (ORF) of ∼3000 amino acids, flanked by highly conserved un-translated regions. The polyprotein is co- and post-translationally processed by host and viral proteases resulting in 10 individual viral proteins, the structural proteins core, E1, E2, and p7 and the non-structural proteins NS2, NS3, NS4A, NS4B, NS5A, and NS5B (2).

HCV establishes a persistent infection meaning it must maintain the viability of virus-infected cells. While the survival of infected cells is critical for viral pathogenesis, the molecular mechanisms by which HCV achieves this still remain largely uncharacterized. In this regard, NS5A is a protein of particular interest as, in addition to its direct role in both viral replication (3) and virion production (4–6), it is known to bind numerous host cell proteins which are involved in cellular signaling pathways (7). NS5A mediates some of these effects via polyproline (PXXP*X*R) motifs, one of which in particular (termed P2) is absolutely conserved in all HCV isolates from all 7 genotypes (8, 9). P*XX*P*X*R motifs form extended helical structures and are present in a plethora of viral and cellular proteins involved in cell signal transduction. They bind to Src homology 3 (SH3) domains found in a range of signal-transducing molecules.

**FIGURE 1.**
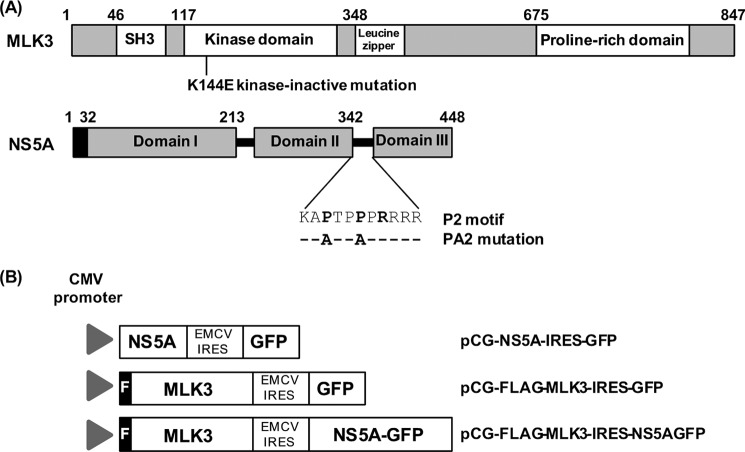
*A*, molecular organization of MLK3 and HCV NS5A. Schematic illustration of the domain structure of the two proteins. Amino acid residue numbers indicated. The sequence of the P2 motif and the mutations introduced to generate the PA2 mutant are illustrated. The black box on NS5A is the N-terminal membrane-associating amphipathic helix. *B*, structure of bicistronic vectors used for transfection in this study. The construction of these vectors is detailed in the “Experimental Procedures.”

Ion channels play a major role in the maintenance of ionic homeostasis. Acute enhancement of voltage-gated K^+^ channel activity can result in the initiation of apoptosis (10). P38 mitogen-activated protein kinases (p38MAPK) are mitogen-activated protein kinases activated in response to stress stimuli, including cytokines and heat shock, that are involved in cell differentiation and apoptosis (11, 12). It was recently demonstrated that in response to oxidative stress, activated p38MAPK phosphorylates the Kv2.1 protein, which mediates an K^+^ efflux with a concomitant induction of apoptosis (10, 13). We previously identified that HCV NS5A inhibits the function of Kv2.1 by inhibiting p38MAPK signaling, an effect mediated through the P2 motif. Accordingly, when the P2 motif was mutated (termed PA2) (Fig. 1*A*) in the context of the HCV isolate JFH1, the virus did not block activation of p38MAPK or Kv2.1, and infected cells were sensitive to oxidant-induced apoptosis (14).

In the present study, we aimed to dissect further the mechanism of NS5A mediated p38MAPK and subsequent Kv2.1 inhibition. We screened a phage-display library expressing the entire complement of human SH3 domains, identifying mixed lineage kinase 3 (MLK3), a serine/threonine protein kinase that functions as a mitogen-activated protein kinase kinase kinase (MAP3K) to activate p38 MAPK, as a NS5A interacting partner. MLK3 is a kinase that activates c-Jun NH_2_-terminal kinase (JNK) and p38MAPK (15). A schematic of the structure of MLK3 is shown in Fig. 1*A*. It is known as a regulator of neuronal apoptosis (16), cytokine-induced apoptosis of hepatoma cells (17), and drug-induced liver injury (18). In this study, we further demonstrate that MLK3 activates Kv2.1 and induces cellular apoptosis, processes suppressed by HCV NS5A in a P2-dependent manner. These data indicate that NS5A counteracts oxidative-stress induced apoptosis and the activation of Kv2.1-mediated K^+^ efflux through the perturbation of MLK3/p38MAPK signaling.

## EXPERIMENTAL PROCEDURES

### 

#### 

##### Cell Culture

Human hepatoma cell lines, Huh7 and Huh7.5, were maintained in Dulbecco's modified Eagle's medium (DMEM) supplemented with 10% fetal bovine serum, 100 units/ml penicillin, 100 μg/ml streptomycin, 1 mm non-essential amino acids, and 2 mm GlutaMax (Invitrogen, Carlsbad, CA). HEK293 Flp-in Tet-on cells were a kind gift from Adrian Whitehouse (University of Leeds, UK) and have been established per the manufacturer's instructions (Invitrogen, Carlsbad, CA).

##### Reagents

2,2′-Dithiodipyridine (DTDP) (Sigma) was dissolved in dimethyl sulfoxide (Hybri-Max, Sigma) and used at 25 μm in the culture medium. Tetracycline (Sigma) was dissolved in ethanol and added to the culture medium to induce protein expression from the tetracycline-regulated promoter at a concentration of 10 ng/ml, unless otherwise noted.

##### Plasmids

pJFH1 was provided by Takaji Wakita (National Institute of Infectious Disease, Japan) and described previously (19). pRG4-Kv2.1 and its corresponding S800A and S800E mutants were provided by Elias Aizenmann (University of Pittsburgh) and described in (13). The cDNAs were used to construct pcDNA5-FRT/TO-Kv2.1 by conventional recombinant DNA techniques. pcDNA-FLAG-MLK3 and the corresponding kinase dead mutant (K144E) were provided by Friedemann Kiefer (Max-Planck Institute, Germany) and described in Ref. 20. For the generation of bicistronic expression vectors expressing different combinations of NS5A and GFP or MLK3 and GFP (Fig. 1*B*), protein coding sequences were amplified by PCR using pJFH1 or pcDNA3-FLAG-MLK3 as template DNA, respectively (all primer sequences available on request). Amplified DNA fragments were digested with XbaI and AscI then cloned into XbaI and MluI digested from a pCG-Nef-IRES-GFP vector (21). To construct pCG-FLAG-MLK3-IRES-NS5AGFP, an in-frame fusion of NS5A and GFP was created by PCR and recloned into the corresponding sites of pCG-FLAG-MLK3-IRES-GFP. The One STrEP-tagged subgenomic replicon clone pSGR-JFH1–5A1ST was described previously (22). The PA2 mutation to disrupt the P2 SH3 binding motif was described previously (23) and was introduced into pSGR-JFH1–5A1ST by conventional molecular cloning techniques. One-STrEP-tagged replicon cell lines and STrEP-tactin affinity column purification were made and performed as described previously (22).

##### Lentiviral Vector System for shRNA Delivery

pCS-RfA-EG and a set of packaging plasmid vectors, including pCMV-VSV-G-RSV-Rev and pCAG-HIVgp, were obtained from Hiroyuki Miyoshi (Riken BioResorce Center, Japan). To construct shRNA expressing lentiviral vectors, a pair of 65-mer oligonucleotides was inserted into BglII and XbaI sites of pENTR4-H1. Oligonucleotide sequences for constructing shGFP and shMLK3 are available upon request. The shRNA encoding insert was confirmed by DNA sequencing. The shRNA expression unit in pENTR4 was inserted into pCS-RfA-EG by Gateway® LR clonase® II (Invitrogen) directed DNA recombination per the manufacturer's recommendation. To obtain lentiviral particles, packaging transfections were performed using Fugene 6 (Promega), as per the manufacturer's recommendations.

##### mRNA Quantitation by qRT-PCR

Total RNA samples were extracted by using Trizol reagent following the manufacturer's instruction. pcDNA3-FLAG-MLK3 vector was linealized by SalI restriction enzyme and treated with mung bean nuclease. Linearized vector was phenol/chloroform extracted for purification and used as template DNA for *in vitro* T7 transcription. Transcribed RNA was purified by phenol/chloroform extraction and examined for their homogeneity prior to use as copy number control samples for quantitative reverse-transcriptase PCR. 100 ng total RNA was combined with 250 ng of random hexamer and subjected to first strand synthesis using superscript II reverse transcriptase. qRT-PCR was performed as described previously in Ref. 22 (primer sequences available upon request).

##### Establishment of HEK293 lines for Conditional Kv2.1 Expression

To establish a stable cell line expressing Kv2.1 under a tetracycline regulated promoter, knock-in transfections were performed as per the manufacturers' instruction. Briefly, HEK293 Flp-in Tet-on cells were seeded into 6-well plates and transfected with pcDNA5 FRT/TO-Kv2.1 together with pOG44 using Trans-it LT1 reagent (Mirus Bio). Forty-eight hours post-transfection, cells were selected with 100 μg/ml hygromycin B for 3 weeks. Colonies were then isolated using cloning cylinders and expanded for further characterization.

##### Indirect Immunofluorescence Microscopy

Cells grown on glass coverslips were washed twice with PBS and fixed with 4% paraformaldehyde in PBS supplemented with 2 mm MgCl_2_ and 1.25 mm EGTA, for 1 h at room temperature. Fixed cells were labeled with primary antibodies overnight in antibody-binding buffer (PBS with 0.2% saponin, 0.2% nonfat dry milk, and 1% bovine serum albumin). After washing cells 4 times in PBS, cells were further incubated with fluorescent secondary antibodies for 4 h, rinsed, and mounted with Prolong gold antifade reagent (Invitrogen).

##### Western Blotting and Immunoprecipitation Analysis

For surface protein labeling on plasma membrane, EZ-Link® Sulfo-NHS-SS-biotin (Pierce) was used as following manufacturer's instruction. Biotin-labeled plasma membrane proteins were collected with streptavidin magnetic beads (New England Biolabs) and subjected to Western blotting analysis. Equal numbers of cells (1 × 10^6^) were subjected for this analysis.

The primary and secondary antibodies used in this study are listed as follows. Sheep anti-NS5A was described previously (24). Anti-FLAG antibody (mouse monoclonal clone M2) was purchased from Agilent technologies. Anti-MLK3 antibody (mouse monoclonal clone D-11) was from Santa Cruz Biotechnology Inc. Anti-GAPDH antibody (mouse monoclonal clone 6C5) was obtained from Abcam. Anti-phospho-p38 MAPK (Thr-180/Tyr-182) clone 12F8 was from Cell Signaling technology. Anti-Kv2.1 antibody (mouse monoclonal clone K89/34) was obtained from NeuroMab. Rabbit anti-phospho Kv2.1 (S800) antibody was provided by Elias Aizenman (University of Pittsburgh). For secondary antibody, peroxidase-conjugated anti-mouse IgG, anti-sheep IgG, and anti-rabbit IgG were obtained from Sigma. Detected protein signal intensities were analyzed by NIH ImageJ software. For immunoprecipitations, Huh7 cells were lysed in 0.25% deoxycholic acid, 0.1% Triton X-100, 150 mm NaCl, 100 mm Tris/HCl pH 8.0. After brief sonification and centrifugation, 0.5 ml of each cleared lysate was incubated with 2 μg of anti-FLAG monoclonal antibodies (clone M2- Agilent) for 1 h at 4 °C. Immuno-complexes were captured with protein G Sepharose beads by incubation at 4 °C for 1 h. Bound immuno-complexes were washed four times with 1 ml of lysis buffer and once with Tris-buffered saline. The band intensities were measured using NIH ImageJ 1.63 software.

##### Electrophysiological Recordings of K^+^ Currents

Cells grown on coverslips were placed on a continuously perfused recording chamber, which was mounted on an inverted fluorescence microscope. Patch pipette resistance was between 4–6 MΩ, and GΩ seals were obtained before proceeding to rupturing the plasma membrane for whole cell recordings. To record K^+^ currents, a series of depolarizing steps (500 ms duration, applied every 5s) from −100 to +60 mV in 10 mV increments, were applied to cells which were otherwise clamped at −70 mV. Voltage-clamp and data acquisition were performed with the use of Axopatch 200A amplifier/Digidata 1200 interface controlled by Clampex 9.0 software (Molecular Devices). On-line leak subtraction was applied using a P/4 protocol, and offline data analysis was performed by Clampfit 9.0 (Molecular Devices). Results were presented as means ± S.E. (*n* = 5, unless otherwise noted), and statistical analysis was performed using unpaired Student's t-tests, where *p* values were indicated in each figure legends. *p* < 0.05 was considered statistical significant. Recordings were performed using the following solutions: Pipette solution; 10 mm HEPES/KOH (pH 7.2), 140 mm KCl, 5 mm EGTA, 2 mm MgCl_2_, 1 mm CaCl_2_, and 10 mm d-glucose. Perfusate buffer; 10 mm HEPES/KOH (pH7.2), 140 mm NaCl, 5 mm KCl, 2 mm MgCl_2_, 2 mm CaCl_2_, and 10 mm d-glucose.

## RESULTS

### 

#### 

##### NS5A Binds to Mixed Lineage Kinase 3 via a Conserved Polyproline Motif

NS5A interacts with a range of host cell proteins to modulate their function and promote virus replication and pathogenesis. In this regard, NS5A contains a proline-rich motif (with the sequence P*XX*P*X*R), designated as P2, which binds to Src homology 3 (SH3) domains (23). This motif is absolutely conserved in all HCV isolates in the Los Alamos database, implying that it must play some role in the virus lifecycle. Paradoxically, mutation of this motif by alanine substitution of the key prolines (termed PA2) has no effect on virus replication (23).

In an attempt to ascribe a function to the P2 motif, we previously reported that HCV attenuates outward K^+^ currents mediated by the Kv2.1 channel in a P2 dependent manner (14), whereby channel activity was found to be impaired by inhibition of p38MAPK phosphorylation of serine 800 (S800) in the C-terminal cytoplasmic domain of Kv2.1. To investigate the molecular mechanism of this effect, we screened a phage display library expressing the entire complement of human SH3 domains (25) to identify putative NS5A interacting partners. This screen led to the identification of mixed lineage kinase 3 (MLK3) (Fig. 1*A*) as a P2-dependent NS5A interacting partner. Interestingly, MLK3 is an upstream activator of p38MAPK, modulating its phosphorylation in response to cytokine and oxidative stress (17, 26, 27). Thus MLK3 was a likely candidate to mediate the NS5A effect on p38MAPK and Kv2.1 activities.

**FIGURE 2.**
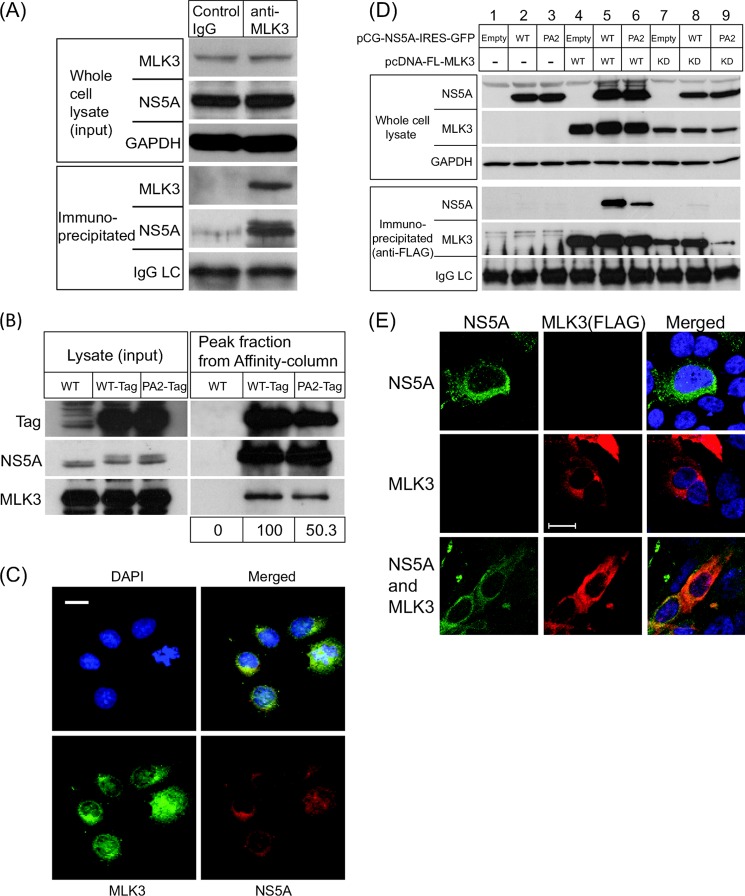
**NS5A binds to MLK3 in a polyproline motif (P2)-dependent manner.** *A*, lysates from Huh7 cells harboring a JFH-1 derived subgenomic replicon (SGR-JFH1) were immunoprecipitated with an anti-MLK3 monoclonal antibody and analyzed by Western blot for the presence of MLK3 and NS5A. The *top panel* shows the lysates and the *bottom panel* the immunoprecipitates, IgG LC: light chain of the immunoprecipitating antibody. *B*, lysates from Huh7 cells harboring an SGR with a One-STrEP-tagged NS5A were purified by affinity column capture with Strep-Tactin beads, prior to Western blotting for the presence of NS5A and MLK3. *C*, SGR-JFH1 cells were analyzed by indirect immunofluorescence with antibodies to NS5A (*red*) and MLK3 (*green*). The merged image shows extensive colocalization of the two proteins. Scale bar, 20 μm. *D*, ectopic expression of NS5A and MLK3, co-immunoprecipitation/Western blotting to demonstrate protein-protein interaction between NS5A and MLK3. Huh7 cells were co-transfected with a combination of plasmid vectors expressing NS5A (wild type or PA2 mutant) and FLAG-tagged MLK3 (wild type or K144E kinase-dead mutant, MLK3 KD) as indicated. Lysates were immunoprecipitated with an anti-FLAG antibody and Western blotted for the presence of NS5A or MLK3. IgG LC: light chain of the immunoprecipitating antibody. *E*, Huh7 cells were transfected with plasmid vectors expressing NS5A (*top row*), FLAG-tagged MLK3 (*middle row*), or both (*bottom row*). Indirect immunofluorescence microscopy reveals colocalization of NS5A and MLK3 in the cotransfected cells.

We sought to confirm this interaction by co-immunoprecipitation. Endogenous MLK3 was immunoprecipitated from lysates of Huh7 cells stably harboring a JFH1-derived subgenomic replicon, Western blot analysis of the immunoprecipitates demonstrated the presence of both MLK3 and NS5A (Fig. 2*A*), while immunoprecipitation with a control IgG did not capture either MLK3 or NS5A. We undertook the reciprocal experiment by affinity capture purification from lysates of Huh7 cells harboring a subgenomic replicon containing a one-STrEP-tag within NS5A. Lysates from wild type (untagged), or tagged replicon cells (NS5A wild type or PA2 mutation) were subjected to STrEP-tactin Sepharose column purification. Comparable amounts of NS5A were expressed in all three cell lines as seen in Fig. 2*B* (*left*), however as expected after affinity capture purification, NS5A was only precipitated and eluted when tagged. MLK3 was co-purified with either wild type or PA2 NS5A, but densitometry revealed that a lesser amount of MLK3 was associated with PA2 NS5A, (Fig. 2*B*, *right*). In support of the coimmunoprecipitation data, indirect immunofluorescence analysis revealed that NS5A and MLK3 were colocalized in perinuclear regions of subgenomic replicon harboring cells (Fig. 2*C*).

To further investigate the requirements for the interaction between NS5A and MLK3, Huh7 cells were transfected with plasmids expressing wild type or PA2 NS5A (genotype 2a JFH-1) and FLAG-tagged MLK3. Ectopic expression of MLK3 leads to autocatalytic self-activation. For this reason, wild type MLK3 appears as multiple bands of different molecular weight (Fig. 2*D*, *lanes 4–6*, *2nd panel*). In contrast, a kinase-inactive mutant of MLK3, where lysine 144 was substituted with glutamic acid (K144E) to eliminate ATP binding, always appeared as a single band (Fig. 2*D*, *lanes 7–9*, *2nd panel*). Intriguingly, NS5A co-precipitated only with wild type MLK3 (*lanes 5* and *6*, *4th panel*), but did not interact with the kinase inactive form of MLK3 (*lanes 8* and *9*). Further to this, when coexpressed with wild type MLK3, but not the K144E mutant, NS5A also exhibited an additional high molecular weight species (*lanes 5* and *6*), consistent with the presence of the hyperphosphorylated (also referred to as p58) NS5A species in subgenomic replicon lysates (see Fig. 2*A*). This suggests that the interaction between MLK3 and NS5A may alter the phosphorylation status of NS5A, either by directly phosphorylating NS5A, or modulating the activity of another (as yet unidentified) NS5A kinase. The interaction of NS5A and MLK3 was significantly impaired by the PA2 mutation (compare *lanes 5* and *6*). In further support of this interaction, we demonstrated by indirect immunofluorescence microscopy that NS5A and FLAG-MLK3 colocalized in transiently transfected cells (*lower panel*, Fig. 2*E*). Our data are consistent with a direct interaction between NS5A and MLK3, mediated in part by the P2 motif of NS5A.

##### NS5A Inhibits MLK3 Induced Apoptosis in a P2 Motif-dependent Manner

**FIGURE 3.**
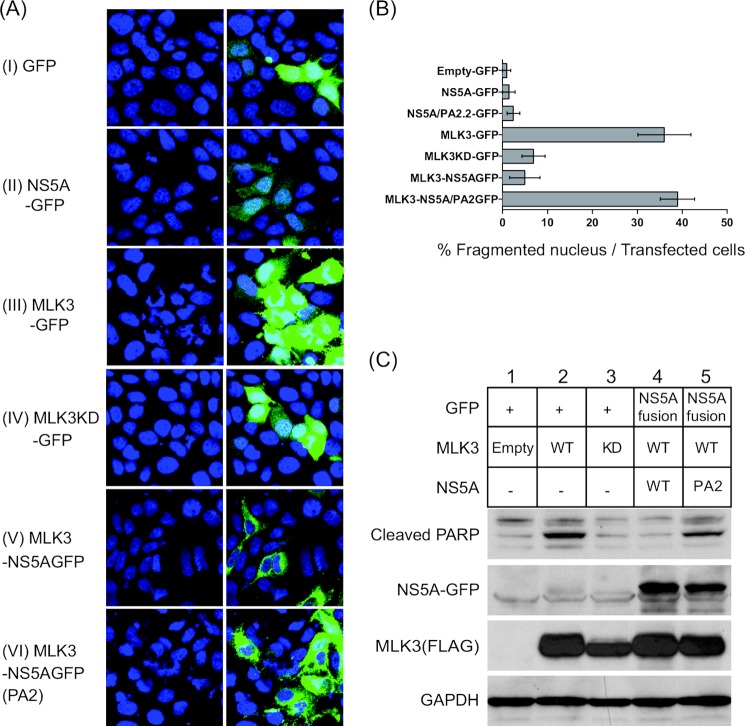
**Exogenous MLK3 expression induces apoptosis.** *A*, Huh7 cells were seeded on coverslips and transfected with the indicated expression vectors. 24 h after transfection, cells were fixed and stained with DAPI. Fluorescence microscopy was performed to visualize transfected cells (GFP fluorescence) and the nucleus (DAPI). *B*, multiple fields were counted (*n* = *4*). GFP-positive cells, more than 60 cells for each sample point, were counted as transfected cells and the percentage of GFP-positive cells showing fragmented nuclei are presented. *C*, Huh7 cells were transfected with the indicated plasmid vectors. Lysates were subjected to Western blotting to detect caspase-cleaved PARP (as a marker of apoptosis), as well as NS5A-GFP and FLAG-MLK3.

We previously demonstrated that the inhibition of Kv2.1 activity by NS5A was accompanied by a concomitant protection of infected cells from oxidative stress-induced apoptosis (14). However, it was not clear whether this protection from apoptosis was due to the block in K^+^ efflux, or some other consequence of the inhibition of p38MAPK signaling. To address this question we next sought to determine the downstream effects of the NS5A-MLK3 interaction. In particular we wished to assess whether MLK3 could mediate the induction of apoptosis and, if so, whether NS5A could abrogate this effect. It had been previously shown that when ectopically overexpressed wild type MLK3 can function as a dominant active kinase via homo-dimerization and subsequent auto-phosphorylation activation, whereas the kinase-inactive K144E mutant functioned as a dominant negative (28). Huh7 cells were therefore transfected with bicistronic constructs expressing MLK3 (either wild type or K144E) and either GFP or a NS5A (wild type or PA2) GFP fusion (Fig. 1*B*). The induction of apoptosis was assessed by the presence of fragmented nuclei at 24 h after transfection. Fig. 3*A* shows that, whereas either GFP or NS5A-GFP expression did not induce apoptosis, as anticipated ectopic MLK3 expression resulted in significant induction of apoptosis. Interestingly, expression of K144E MLK3 did not induce apoptosis suggesting that MLK3-mediated cell death is mediated by the kinase activity of MLK3. When MLK3 was expressed together with wild type NS5A-GFP, MLK3-mediated cell death was inhibited. Importantly, this inhibition was not observed upon co-expression of PA2 NS5A (Fig. 3*A*-VI). These results were quantified and expressed as an apoptotic cells in the GFP positive population (Fig. 3*B*). To confirm the induction of apoptosis by MLK3, we utilized an alternative assay for the caspase-3 mediated cleavage of poly[ADP-ribose]polymerase (PARP), a well characterized apoptosis effector molecule. Wild type MLK3 induced strong activation of PARP (Fig. 3*C*, *lane 2*) whereas this effect was abrogated by the K144E mutation of MLK3 (Fig. 3*C*, *lane 3*). Cotransfection of wild type NS5A, but not the PA2 mutant, blocked the appearance of cleaved PARP (*lanes 4* and *5*). Taken together, these observations suggest that NS5A interacts with MLK3 and inhibits its kinase activity and/or accessibility to substrate proteins, thus blocking MLK3-induced apoptosis.

##### NS5A Suppresses Outward K^+^ Current by Inhibiting the Kv2.1 Channel

**FIGURE 4.**
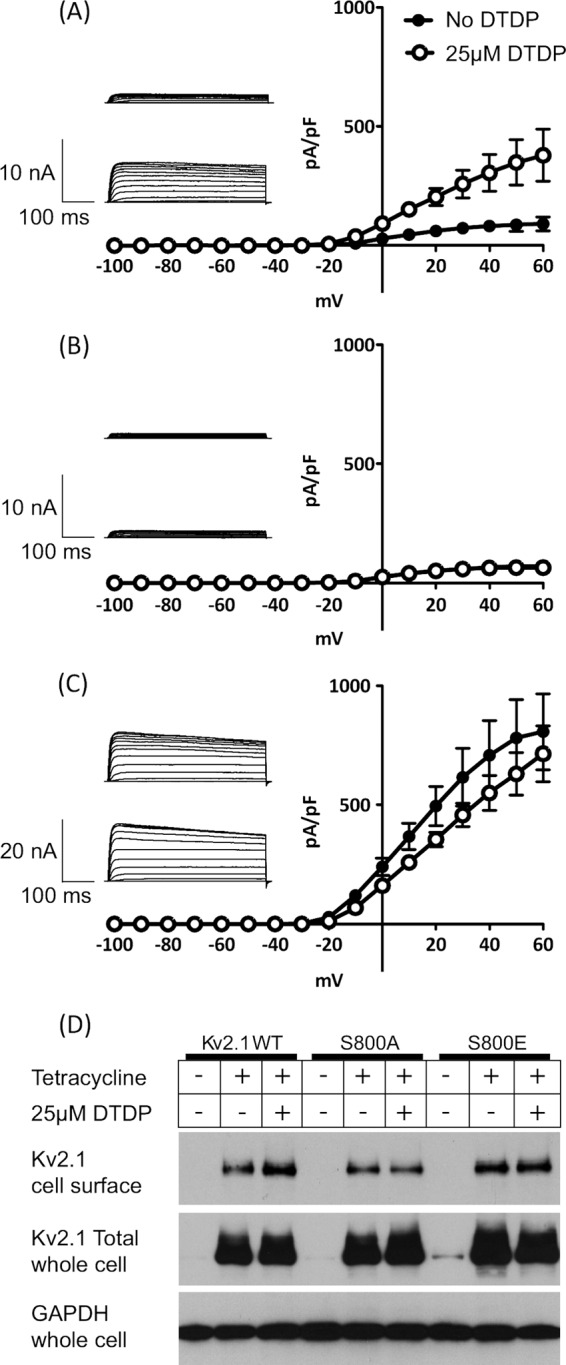
**Kv2.1 ion channel activity is regulated by serine 800 phosphorylation.** Whole-cell patch clamp recordings of Kv2.1 K^+^ currents. HEK293 cell lines expressing wild type Kv2.1 (*A*), S800A (non-phosphorylatable mutant: *B*) or S800E (phosphomimetic mutant: *C*) were subjected to whole cell patch clamp in the presence (○) or absence (●) of the oxidant DTDP (*n* = 3). *Insets* show representative traces of K^+^ currents in patch-clamp recordings obtained by step depolarizations applied from a holding potential of −70 mV to between −100 mV and +60 mV, in 10-mV increments. *Top*; trace without DTDP stimulation, below: with DTDP stimulation (*D*) HEK293 Kv2.1 cells were incubated with tetracycline to induce Kv2.1 expression, and/or DTDP to induce oxidative stress. Proteins expressed on the plasma membrane of HEK293 Kv2.1 cells were biotin-labeled and harvested samples were probed by Western blot with anti-Kv2.1 antibody (*top panel*). Whole cell lysates were also probed with the total Kv2.1 (*middle*). Comparable amount of Kv2.1 proteins were expressed following addition of tetracycline. GAPDH detection was performed as a control (*bottom*).

As we previously reported, the inhibition of both apoptosis and the activity of the Kv2.1 channel were dependent on the P2 motif within NS5A (14). Taken together with the results shown in Figs. 2 and 3, we therefore considered that MLK3 might also contribute to Kv2.1 regulation. To test this, and further elucidate the mechanism by which NS5A inhibited Kv2.1 activity, we established a novel model cell line for electrophysiological measurement of Kv2.1 activity and its regulation. Stable HEK293 cell lines expressing either wildtype Kv2.1, a non-phosphorylatable S800A, or a phosphomimetic S800E mutant, under the control of tetracycline-regulatable promoter were produced. For cells expressing wild type Kv2.1, we were able to record a typical voltage gated outward K^+^ current upon tetracycline induction (Fig. 4*A*) that could be stimulated by oxidant (DTDP) treatment. Currents were augmented 3.5–4 fold by DTDP treatment over the voltage range +10 mV to +60 mV.

As expected, the S800A mutant showed an attenuated current density that was non-responsive to oxidant treatment, (Fig. 4*B*), as this mutant lacks the key phosphorylation residue for trafficking and membrane insertion following stress activation. By contrast the S800E mutant exhibited constitutive activity, giving rise to large outward K^+^ currents regardless of DTDP stimulation (Fig. 4*C*). These data confirm that S800 phosphorylation of Kv2.1 is a key event in the control of channel activity in the inducible cell lines employed in this study. These data correlated with the cell surface expression of Kv2.1 as detected by biotinylation of cell surface proteins followed by streptavidin-agarose precipitation and Western blotting for Kv2.1 (Fig. 4*D*, *top panel*). However, it is noteworthy that, by Western blotting with an antibody specific for the S800 phosphorylated form of Kv2.1, we did not observe a concomitant increase in S800 phosphorylation following DTDP treatment. Clearly there is a high level of basal S800 phosphorylation in HEK293 cells and other events such as Kv2.1 Y124 phosphorylation might be playing a role in cell surface expression of active Kv2.1. Our data are consistent with a key role for S800 in the oxidant-induced cell surface expression and activity of Kv2.1, because both the S800A and S800E mutants are non-responsive to DTDP (Fig. 4, *B* and *C*).

**FIGURE 5.**
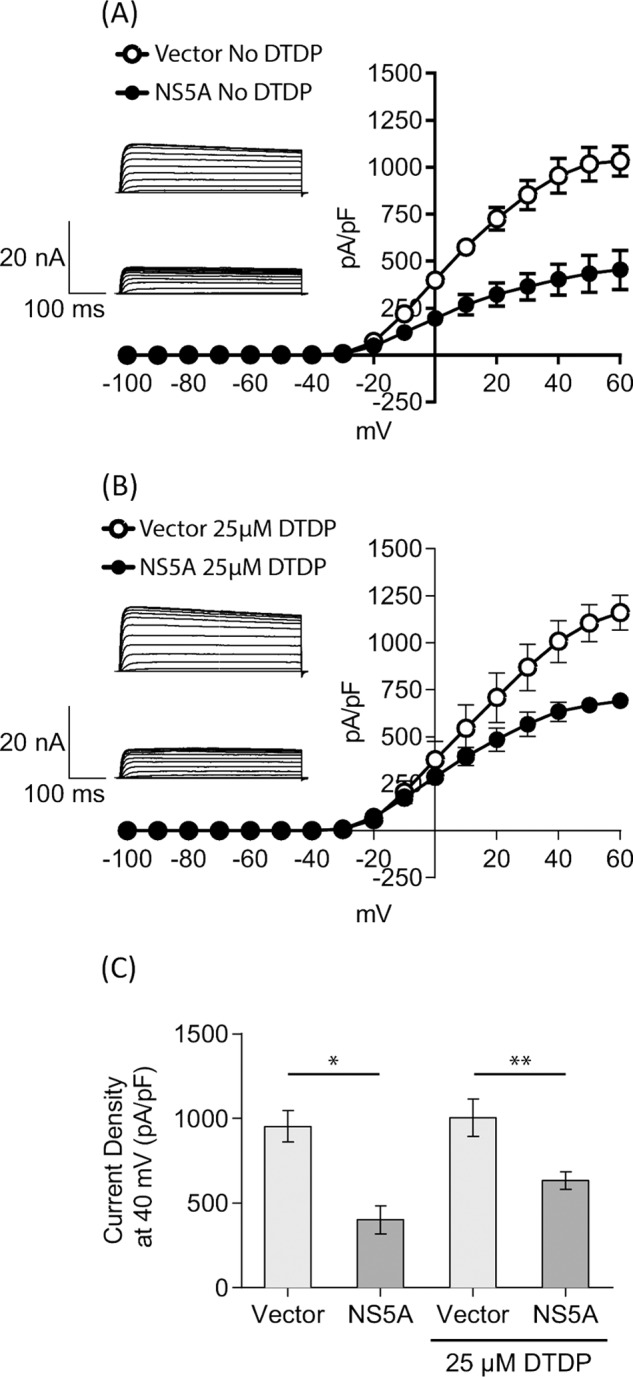
**NS5A suppresses Kv2. 1 channel activity.** HEK293 cells expressing Kv2.1 were transfected with bicistronic vectors encoding NS5A and GFP, or GFP alone (shown in Fig. 1*B*). At 24 h after transfection, Kv2.1 expression was induced by tetracycline treatment for 12 h, prior to electrophysiological analysis either without (*A*), or following (*B*) of DTDP stimulation (*n* = 6). *Insets* show representative traces of outward K^+^ currents in patch-clamp recordings. *Top*; control trace in GFP-expressing cells, below: trace from cells expressing NS5A and GFP. *C*, comparison of current density measurements from *A* and *B* at +40 mV. *, *p* < 0.002, **, *p* < 0.02, unpaired *t* test.

We previously demonstrated that HCV genotype 1b NS5A fused to GFP was able to inhibit basal Kv2.1 activity in Huh7 cells (14). To confirm that this phenotype was also a property of the JFH-1 NS5A, and to test the requirement for S800 phosphorylation, we transfected the tetracycline inducible HEK293 Kv2.1 cell lines with a bicistronic vector encoding NS5A and GFP, (Fig. 1*B*). Following the addition of tetracycline, whole cell recordings were performed from GFP-positive cells. JFH-1 NS5A also effectively suppressed channel activity by up to 50% either without or with oxidative stimuli (Fig. 5, *A* and *B*, respectively). As shown in Fig. 5*C*, the current densities measured at +40 mV were reduced by ∼50% in NS5A-expressing cells in both cases.

##### Endogenous MLK3 Is Required for S800 Phosphorylation Activation of Kv2.1

**FIGURE 6.**
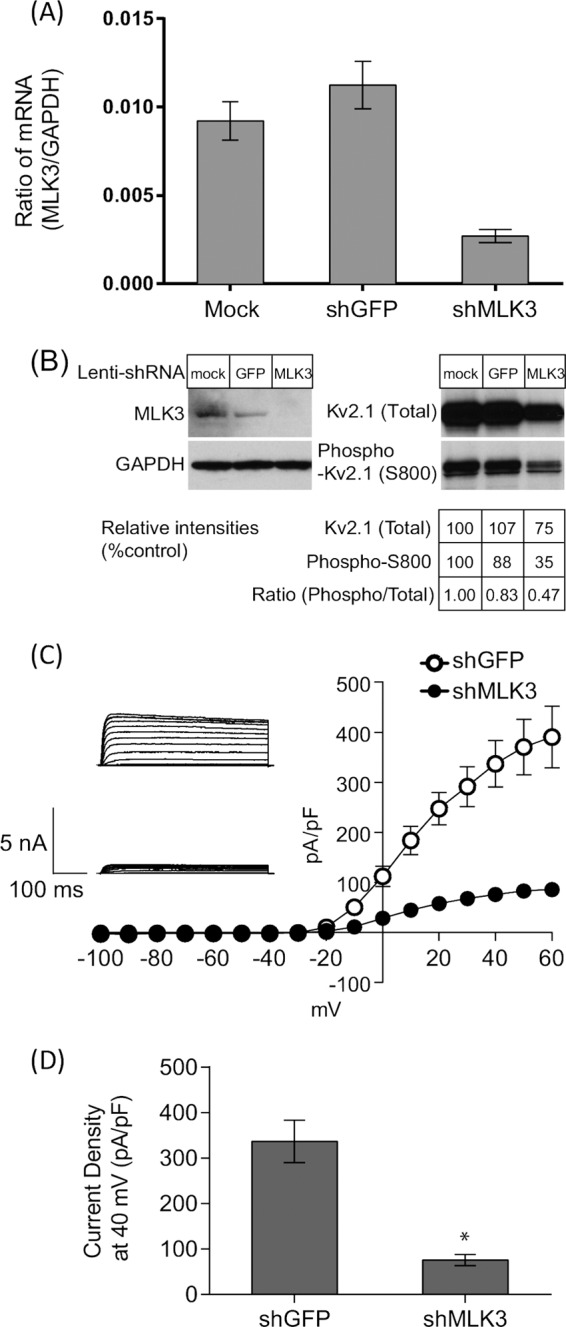
**Endogenous MLK3 contributes to phosphorylation activation of Kv2. 1 HEK293 cells expressing Kv2.1 were infected with lentiviral vectors expressing shRNA targeting either MLK3 or GFP.** After 6 days, cells were analyzed by quantitative-RT-PCR (*A*) or Western blotting (*B*). *A*, absolute quantitation of both MLK3 and GAPDH mRNA was performed and shown as a ratio. *B*, Western blotting with indicated antibodies. Cells were infected as follows: *lanes 1*: control (empty) lentiviral vector infection, *lanes 2*: lentiviral vector expressing shGFP, *lanes 3*: lentiviral vector expressing shMLK3. GAPDH was detected as a control. Western blots were quantitated by densitometry to show a reduction in Kv2.1 S800 phosphorylation following MLK3 knock-down. *C*, electrophysiological measurements of Kv2.1-mediated outward K^+^ currents from shRNA-expressing cells. *D*, comparison of current density measurements at +40 mV from shRNA-expressing cells. *, *p* < 0.001, unpaired *t* test.

To investigate if MLK3 was involved in regulating Kv2.1 channel activity, we performed shRNA-mediated knock down of MLK3. Tetracycline-inducible HEK293 Kv2.1 cells were infected with lentiviral vectors to deliver shRNA targeting MLK3 (shMLK3) or targeting GFP (shGFP) as a control. RNA interference achieved ∼75% reduction of MLK3 in transcriptional level (Fig. 6*A*), leading to a significant reduction of MLK3 protein expression (Fig. 6*B*, *lane 3*, *top panel*). Interestingly, impaired MLK3 expression caused an attenuation of Kv2.1 S800 phosphorylation (Fig. 6*B*, *lane 3*, *2nd panel*). In accordance with this observation, K^+^ outward currents carried by Kv2.1 were measured by whole cell patch clamp from MLK3 knock down cells, and were found to be reduced by 80%, compared with control (Fig. 6, *C* and *D*). Altogether, these results imply that MLK3 can regulate the function of the Kv2.1 channel. Therefore, NS5A binding to, and inhibition of, MLK3 could also affect Kv2.1 channel activity.

##### MLK3 Activation of Kv2.1 Is Inhibited by NS5A

**FIGURE 7.**
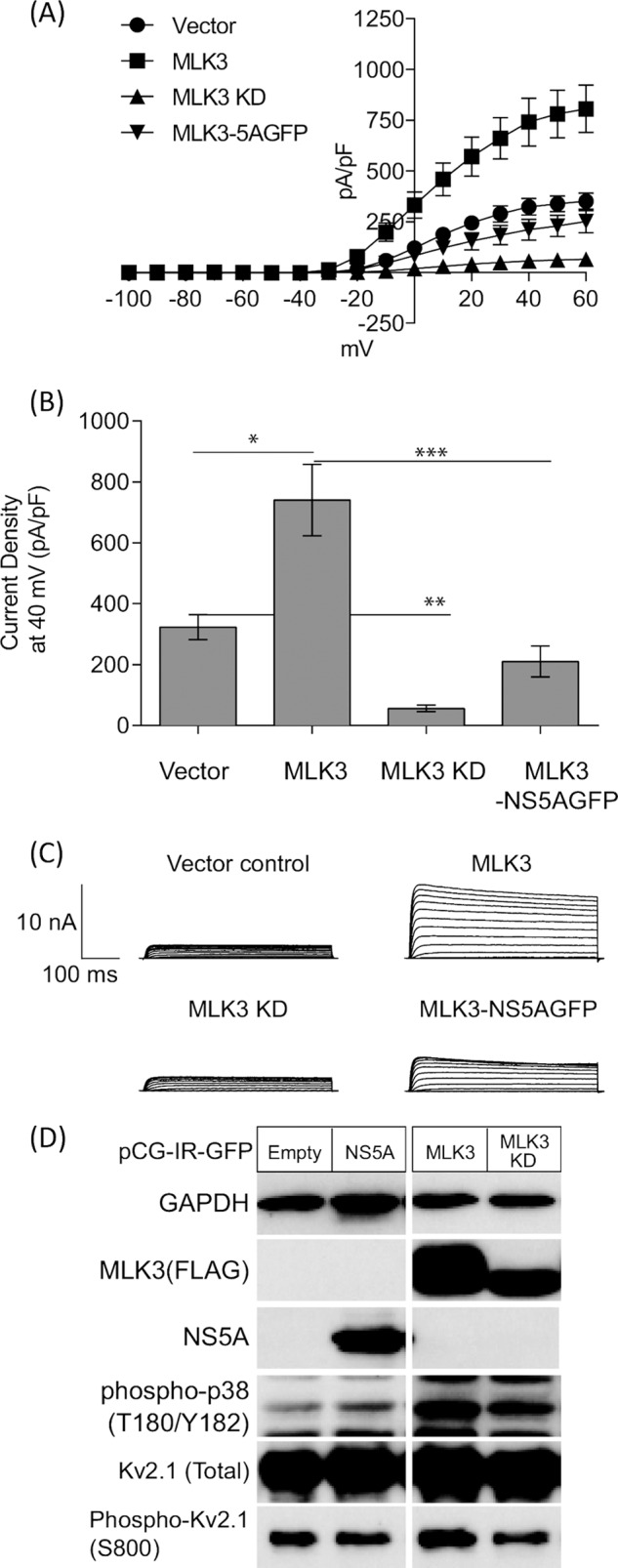
**MLK3 acts as an upstream regulator of Kv2. 1 channel via Ser800 phosphorylation.** *A*, HEK293 cells expressing Kv2.1 were transfected with bicistronic vectors expressing MLK3 (wild type or K144E) together with GFP or NS5A-GFP as shown in Fig. 1*B*. 16 h post-transfection, Kv2.1 expression was induced by tetracycline treatment, and 8 h later GFP-positive cells were identified by fluorescent microscopy and subjected to electrophysiological recordings. Overexpression of MLK3 wild type augments Kv2.1 channel activity (■), whereas, the K144E kinase-inactive mutant suppressed channel activity (▴). Co-expression of NS5A-GFP abrogated the MLK3 mediated enhancement of Kv2.1 activity (▾). *B*, comparison of current density measurements at +40 mV. *, *p* < 0.02, **, *p* < 0.001, and ***, *p* < 0.005, unpaired *t* test. *C*, representative traces of outward K^+^ currents in patch-clamp recordings. *D*, lysates from HEK293 cells expressing Kv2.1 wild type were analyzed by Western blotting with the indicated antibodies. Cells were transfected as follows: *lane 1*: empty vector, *lane 2*: NS5A-IRES-GFP, *lane 3*: MLK3 wild type-IRES-GFP, and *lane 4*: MLK3 K144E-IRES-GFP.

To investigate whether MLK3 is directly involved in p38MAPK mediated Kv2.1 activation, we transfected tetracycline inducible HEK293 Kv2.1 cells with a bicistronic vectors expressing either wild type or the K144E kinase-inactive mutant of MLK3, together with GFP, and performed whole-cell patch clamp recordings from GFP-positive cells. Exogenous expression of wild type MLK3 significantly enhanced Kv2.1 channel activity (Fig. 7, *A–C*), while the K144E kinase-inactive mutant showed a dominant negative effect in terms of phosphorylation activation of Kv2.1. To further demonstrate the activation of Kv2.1 in response to MLK3 overexpression, we investigated both p38MAPK activation and Kv2.1 phosphorylation in cells expressing wild type and K144E MLK3. Western blotting analysis demonstrated that MLK3 overexpression led to the activation of both p38MAPK and subsequent Kv2.1 S800 phosphorylation (Fig. 7*D*, *lane 3*), which was not observed following transfection of K144E MLK3 (Fig. 7*D*, *lane 4*). As NS5A interacts with MLK3 (as shown in Fig. 1), we hypothesized that the inhibition of Kv2.1 activity by NS5A may be mediated through MLK3 inhibition. Accordingly, when cells were transfected with a bicistronic vector expressing wild type MLK3 and an NS5A-GFP fusion protein, the levels of Kv2.1 activity were significantly reduced, suggesting that NS5A inhibits the stimulatory effects of MLK3 on Kv2.1 activity (Fig. 7, *A* and *B*).

## DISCUSSION

**FIGURE 8.**
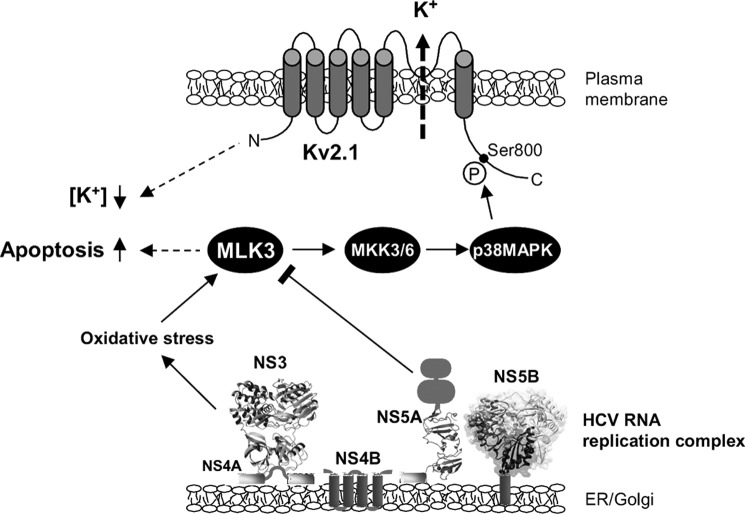
**A model of NS5A interference with MLK3/p38/Kv2.1 signaling.** HCV infection and RNA replication triggers the elevated production of reactive oxygen species (*ROS*), which activates MLK3, leading to activation of p38MAPK. In turn p38MAPK phosphorylates serine 800 of Kv2.1, which is a key phosphorylation event involved in the insertion of Kv2.1 into the plasma membrane. The upsurge in the efflux of K^+^ lowers the intracellular concentration of K^+^, causing an alteration of ionic homeostasis. At the same time MLK3 stimulates the induction of apoptosis. In this study, we show that NS5A inhibits MLK3 activation, thereby blocking both the phosphorylation and activation of Kv2.1, and MLK3-mediated apoptosis.

In this study we set out to investigate the molecular mechanism by which the HCV NS5A protein inhibits the activity of the Kv2.1 potassium channel. This investigation led us to the observation that NS5A interacts with, and inhibits, the MAP3K MLK3. This has a number of potential functional consequences, two of which we have investigated further. A schematic model of the effects of NS5A on MLK3 and the downstream consequences is presented in Fig. 8. First, inhibition of MLK3 abrogates the well-characterized pro-apoptotic role of this kinase. MLK3 was first shown to be pro-apoptotic nearly a decade ago (17), and more recently has been shown to be involved in apoptosis in both pancreatic β-cells (29) and hepatocytes (18) where it was shown to contribute to acetaminophen-induced oxidative stress and hepatotoxicity. Intriguingly, MLK3 was recently shown to be activated by the hepatitis B virus X protein (HBx) leading to increased apoptosis in cultured HepG2 cells (30). In relation to HCV infection, inhibition of MLK3 is therefore potentially important to allow the survival of infected cells and the establishment of a persistent infection. In this regard it is noteworthy that HCV causes elevated intracellular ROS production mediated by iron overload (31), deregulation of ER Ca^2+^ storage (ER stress) (32), and direct mitochondrial ROS production (33). Elevated ROS production can in turn suppress HCV replication (34) and cause a range of pathological features including apoptosis, steatosis, DNA damage, and tumorigenesis (35).

It is important to note that as well as inhibition of MLK3, NS5A targets other cellular pathways to that would potentially block apoptosis and contribute to tumorigenesis. In this regard both we (36, 37), and others (38, 39), have demonstrated that NS5A activates the proto-oncogene β-catenin, leading to its stabilization and stimulating β-catenin-dependent transcription. Intriguingly, MLK3 has been shown to inhibit β-catenin-dependent transcription while paradoxically stabilizing β-catenin (40). It is therefore likely that NS5A could utilize multiple mechanisms to stimulate β-catenin, both by directly binding to it and indirectly by inhibiting MLK3. Clearly, HCV must benefit from the stimulation of β-catenin activity as it has evolved multiple mechanisms to ensure that this occurs during infection.

The second consequence of MLK3 inhibition is that there is a concomitant inhibition of p38MAPK-mediated phosphorylation of the K^+^ channel; Kv2.1. Kv2.1 channel activity has been associated with the induction of apoptosis in neurons (10, 13, 41), and indeed recent observations suggest that NS5A is able to block Kv2.1-mediated apoptosis in cultured rat neurons (42). However, in the present study, we were not able to observe elevated levels of apoptosis in either HEK293 or Huh7 cells following overexpression of Kv2.1 (data not shown). We conclude therefore, that the induction of apoptosis we observed previously in Huh7 cells treated with DTDP (14) was coincident with stimulation of Kv2.1 activity, and not dependent upon the latter. Both the induction of apoptosis and the stimulation of Kv2.1 activity could be inhibited by NS5A in a P2 motif-dependent manner. In the present study we show that both of these effects can be explained by interactions between NS5A and MLK3. Although alanine substitution of the conserved prolines in the P2 motif does not affect viral fitness or replication (23), the same mutation caused a greater sensitivity to oxidative stress-induced apoptosis (14). Additionally, the P2 motif was shown to be important for the establishment of infection in the chimpanzee model (43). Coupled with our previous observation that primary hepatocytes were much more sensitive to oxidative stress than Huh7 (14), we propose that the P2 motif may play a role in virus persistence by blocking the induction of apoptosis in infected hepatocytes. This could explain the absolute conservation of this motif in all HCV isolates.

As mentioned above, a recent study revealed that NS5A of a different genotype (1b) effectively blocked Kv2.1 mediated currents without interfering with S800 phosphorylation but by suppressing Y124 phosphorylation, which is mediated by c-Src kinase (42). The data discrepancy may be due to different experimental conditions (*e.g.* cell type), although it is still noteworthy to mention that NS5A targets c-Src kinase (44). A recent report (45) demonstrated that S440 and S537 of Kv2.1 are substrates of AMP-activated protein kinase (AMPK). Since we have shown that AMPK activation was significantly impaired by HCV infection (46), it will be of great interest to see how Kv2.1 phosphorylation by AMPK is affected during either HCV infection or NS5A expression. Furthermore, a body of etiological evidence shows that HCV infection causes a range of metabolic disorders in patients, for instance fatty liver and insulin resistance. Future studies will hopefully establish how viral suppression or deregulation of Kv2.1 could contribute to such metabolic alterations caused by HCV.

Overall this study demonstrates that the SH3-binding P2 motif of NS5A can bind MLK3 to both suppress oxidative stress induced outward K^+^ currents, and inhibit host cell apoptosis. This may facilitate the maintenance of long term viral infection and in turn may enable HCV to establish a persistent infection. Understanding the mechanisms of NS5A modulation of MLK3 and Kv2.1 provides a target for the development of novel antivirals that may trigger apoptosis in HCV infected hepatocytes.

## Footnotes

4 The abbreviations used are:

HCV: hepatitis C virus
NS: non-structural
MLK: mixed lineage kinase
DTDP: dithiodipyridine
SH: Src homology.
